# Gene therapy for lysosomal storage disorders: recent advances for metachromatic leukodystrophy and mucopolysaccaridosis I

**DOI:** 10.1007/s10545-017-0052-4

**Published:** 2017-05-30

**Authors:** Rachele Penati, Francesca Fumagalli, Valeria Calbi, Maria Ester Bernardo, Alessandro Aiuti

**Affiliations:** 1 Unit of Pediatric Immunohematology and Stem Cell Program, IRCCS San Raffaele Scientific Institute, Milan, Italy; 2 San Raffaele Telethon Institute for Gene Therapy (SR-TIGET), IRCCS San Raffaele Scientific Institute, Milan, Italy; 3 Department of Neurology, IRCCS San Raffaele Scientific Institute, Milan, Italy; 4 Vita Salute San Raffaele University, Milan, Italy

**Keywords:** Lysosomal storage diseases, Transplantation, Gene therapy

## Abstract

Lysosomal storage diseases (LSDs) are rare inherited metabolic disorders characterized by a dysfunction in lysosomes, leading to waste material accumulation and severe organ damage. Enzyme replacement therapy (ERT) and haematopoietic stem cell transplant (HSCT) have been exploited as potential treatments for LSDs but pre-clinical and clinical studies have shown in some cases limited efficacy. Intravenous ERT is able to control the damage of visceral organs but cannot prevent nervous impairment. Depending on the disease type, HSCT has important limitations when performed for early variants, unless treatment occurs before disease onset. In the attempt to overcome these issues, gene therapy has been proposed as a valuable therapeutic option, either ex vivo, with target cells genetically modified in vitro, or in vivo, by inserting the genetic material with systemic or intra-parenchymal, in situ administration. In particular, the use of autologous haematopoietic stem cells (HSC) transduced with a viral vector containing a healthy copy of the mutated gene would allow supra-normal production of the defective enzyme and cross correction of target cells in multiple tissues, including the central nervous system. This review will provide an overview of the most recent scientific advances in HSC-based gene therapy approaches for the treatment of LSDs with particular focus on metachromatic leukodystrophy (MLD) and mucopolysaccharidosis type I (MPS-I).

## Introduction

Lysosomal storage diseases (LSDs) are rare inherited metabolic disorders characterized by a dysfunction in lysosomes. LSDs encompass approximately 70 genetically distinct diseases, with a collective incidence of 1:5000 live births (Vitner et al 2015). Lysosomes are intracellular organelles containing more than 60 hydrolytic enzymes fundamental for macromolecule degradation (Neufeld 2001), such as glycolipids, oligosaccharides, proteins and glycoproteins. These enzymes, produced in the endoplasmic reticulum (ER), are sorted as inactive pro-enzymes from the Golgi apparatus to lysosomes. This trafficking is regulated by a specific marker present on these hydrolytic enzymes, mannose-6-phosphate (M6P), which is recognized by its intracellular M6P receptor (M6PR) and carried to lysosomes. A fraction of these pro-enzymes escapes the lysosomal sorting pathway, is secreted into the extra-cellular space and may be taken up via cell surface M6PR by the producing cell or by other neighbouring cells, giving rise to the well-known phenomenon of “cross-correction” (Neufeld 2001). Deficiency of lysosomal enzymes leads to an accumulation of waste materials, causing cellular dysfunction, alteration of cell morphology, impaired autophagy, oxidative stress, neuroinflammation and impaired organ function in the brain, bones, viscera and connective tissue (Neufeld 1991). LSDs can be classified based on the nature of the primary stored material, namely into mucopolysaccharidoses, mucolipidoses, glycoproteinoses, sphingolipidoses, oligosaccharidosis and glycogen storage diseases (Futerman and van Meer 2004). Recent understanding of the molecular basis of these diseases has allowed a classification based on the molecular defect, subdividing LSDs into disorders due to: (i) non-enzymatic lysosomal protein defects; (ii) transmembrane protein defects (transporters and structural proteins); (iii) lysosomal enzyme protection defects; (iv) post-translational processing defects of lysosomal enzymes; (v) trafficking defects in lysosomal enzymes; and (vi) polypeptide degradation defects (Filocamo and Morrone 2011). Another interesting classification subdivides LSDs based on nervous system involvement. This spectrum of diseases represents a serious therapeutic challenge, causing progressive neuronal dysfunction and death. For most neuronal LSDs, the neurological involvement can be secondary to substrate accumulation in neurons or in adjacent tissue, although in some cases, the neuronal dysfunction is independent from the storage burden (Wraith 2002, Jeyakumar et al 2005).

This review will focus in particular on two autosomal recessive disorders, mucopolysaccharidosis type I (MPS-I) (OMIM # 607014), caused by a deficiency of the enzyme alpha-L-iduronidase, and metachromatic leukodystrophy (MLD, OMIM **#** 250100), due to deficiency of arylsulfatase A (ARSA) enzyme, as models of LSD with nervous system involvement, for which different gene therapy (GT) approaches have been developed in the last few years. As described by Wraith et al (Je 2004), it is well recognized that LSDs are not simply caused by an accumulation of storage materials but result also from an impairment in complex cell-signalling mechanisms. In fact, the dysfunction of a wide variety of enzyme and non-enzyme proteins compromising the lysosomal system determines a pathogenic cascade responsible for disease progression and clinical manifestations. Although all LSDs have similar underlying pathogenic mechanisms, specific features characterize every disease, with variable visceral and neuronal involvement, due to variable expression of various enzymes in different tissues. Furthermore, phenotypic heterogeneity between family members carrying identical mutations has been reported. Most therapeutic strategies for LSDs have tried to correct the biochemical defect by providing an exogenous supply of functional wild-type enzyme, taking advantage of the cross-correction mechanism. Enzyme replacement therapy (ERT) consists in the administration of the recombinant enzyme, usually through the intravenous (iv) route, and it has been exploited in different LSDs, like Pompe disease (Chakrapani et al 2010), type I Gaucher disease (Gonzalez et al 2013), Fabry disease (Alfadhel and Sirrs 2011) and MPS, type I (Laraway et al 2016), II (da Silva et al 2016), IVA (Tomatsu et al 2015), VI (Harmatz and Shediac 2017) and VII (Sands 2014). ERT with parenteral administration of a purified recombinant pro-enzyme has been exploited in different attenuated MPS-I variants (Barranger and O'Rourke 2001, Eng et al 2001, Kakkis et al 2001); however, the Blood Brain Barrier (BBB) severely limits the access of the enzyme (Kobayashi et al 2005), greatly reducing the impact of this strategy both on CNS and peripheral nervous system (PNS). Thus, ERT is not recommended as a sole treatment for MPS-I patients, except in the preparatory periods before allogenic transplantation is scheduled.

**Fig. 1:**
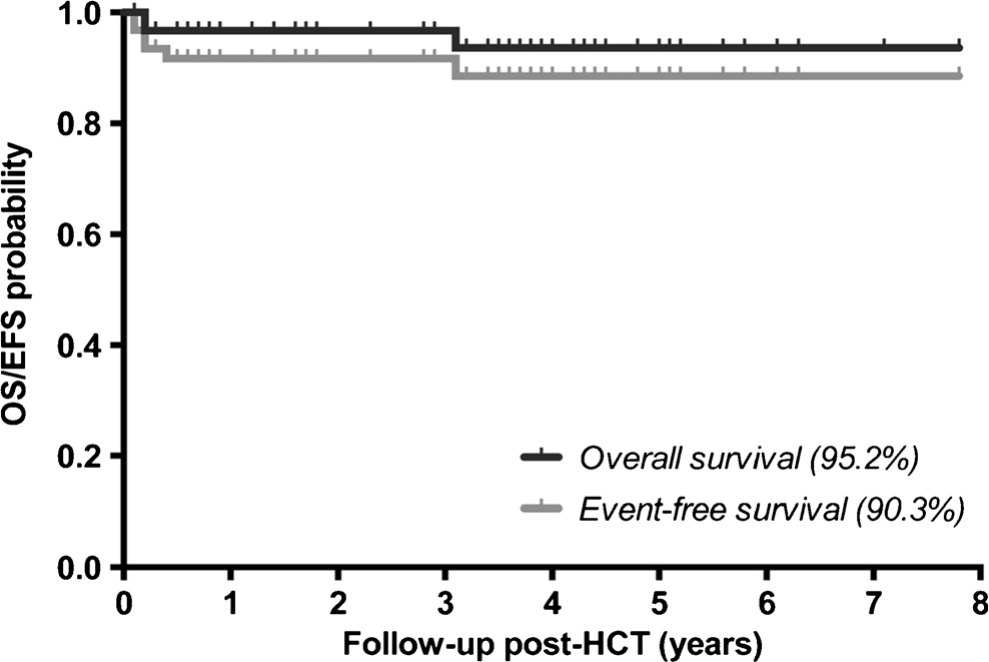
Overall survival and event-free survival after allogeneic HSCT in MPS-I (Aldenhoven et al 2015a)

Considering that intravenous ERT has not been demonstrated to be efficacious in controlling CNS disease manifestations, the BBB limitation has been addressed with different routes of administration, including intra-cerebroventricular or intra-thecal ERT agent delivery and trials are still ongoing to prove its efficacy. In particular, a phase I/II multicentre open-label dose escalation study started in 2011 exploiting the intra-thecal delivery of a biological recombinant of human arylsulfatase A (HGT-1110), for a total of 38 weeks (20 injections) in children with MLD (ClinicalTrials.gov Identifier: NCT01510028). This multi-centric study has now completed the recruiting phase and results are still pending. Unfortunately, one limitation of this approach is the development of an immune response directed to the enzyme, with consequent enzyme inactivation (Matzner et al 2008). This suggests the necessity of an immunosuppressive treatment or of a tolerance induction towards the enzyme to obtain therapeutic benefits from ERT (Matzner et al 2009). Over the past 25 years, thousands of allogeneic haematopoietic stem cell transplants (HSCTs) have been performed worldwide for patients with a wide range of different LSDs. Allogeneic HSCT was firstly exploited in MPSs and over the past two decades, more than 500 HSCT have been performed in MPS-I. In 2005, new international guidelines for HSCT in MPS patients were developed based on a European predictor analysis (Aldenhoven et al 2015a). The group of Aldenhoven (Aldenhoven et al 2015b) and colleagues treated 62 patients, of which 56 were MPS-I patients. The results showed excellent transplant outcomes in terms of overall survival (95.2%) and event-free survival (90.3%) (Fig. 1), confirming that HSCT can significantly ameliorate the clinical course of patients, although leaving a significant residual disease burden (Aldenhoven et al 2015b), mainly on the nervous and bone tissues. In fact, the neurodevelopmental prognosis of patients with MPS-I after HSCT has been demonstrated to be determined by the degree of damage of the CNS, which occurred before the transplant. In fact, HSCT was poorly effective in patients with overt neurological symptoms or in those with early onset or aggressive infantile forms. The likely reason for this limited efficacy is probably related to the slow pace of replacement of fixed tissue macrophages/histiocytes and microglial populations by the transplanted haematopoietic cell progeny as compared to the rapid progression of the primary disease.

In the case of MLD, although HSCT has been reported to provide some benefit, its effectiveness in ameliorating the long-term prognosis of the disease has yet to be proved (Rosenberg et al 2016). The outcomes of a recent study reporting result of a cohort of late infantile (LI) and juvenile patients treated with umbilical cord blood (UCB) transplantation are more promising (Martin et al 2013). This study indicated that early onset patients transplanted after symptom onset did not benefit from the transplant procedure, which was associated with 25–30% mortality rate (Martin et al 2013, Boucher et al 2015). This was also shown by Boucher et al (Boucher et al 2015) who analysed transplantations performed on 40 MLD patients, of which four (10%) were LI, 27 (67%) juvenile and nine (23%) adult MLD. Although most patients ultimately experienced neurologic decline following transplant, the authors showed how HSCT has a more favourable impact on cognitive capabilities compared to motor dysfunction. Furthermore, Van Rappard and colleagues recently showed similar clinical findings on a cohort of 13 MLD patients (2 LI, 5 juvenile and 6 adult) treated in a pre- or early symptomatic phase (IQ > 70 and absence of gross motor impairment) (van Rappard et al 2016). These data indicate that HSCT does not have a clear benefit in LI MLD patients (regardless of symptom status at the time of transplant) or in symptomatic MLD patients, in whom transplantation cannot prevent CNS involvement and damage. Outcomes of HSCT in juvenile patients treated in a pre- or very early symptomatic phase was more favourable but greatly heterogeneous in terms of disease stabilization.

Based on these reported results, HSCT has been proposed as a therapeutic option in early juvenile patients when performed at a pre-symptomatic stage with a long “therapeutic window” (Kehrer et al 2011) (defined as the time from treatment to the expected disease onset). Such patients may be identified through the diagnosis of MLD in an older affected sibling. However, even in this favourable scenario, a longer-term follow up in a larger number of patients is needed to demonstrate the sustained benefit of HSCT. In conclusion, HSCT outcome appears to be influenced by two key variables: i) the disease variant of the patient and ii) the stage of the disease at time of transplant. In particular, HSCT appears to be effective in stabilizing disease progression in early symptomatic late onset patients at long-term follow-up. This differential outcome is likely due to the time required for transplantation to achieve disease stabilization, which is estimated to take as long as 12 or more months. Thus, the more severe and rapidly progressive the phenotype and the longer the interval from onset of first obvious symptoms to HSCT are, the poorer the outcome is. Based on these considerations, the aim of developing alternative therapies for LSDs appears to be not only justified, but also desirable in ethical terms.

## Gene therapy

**Fig. 2:**
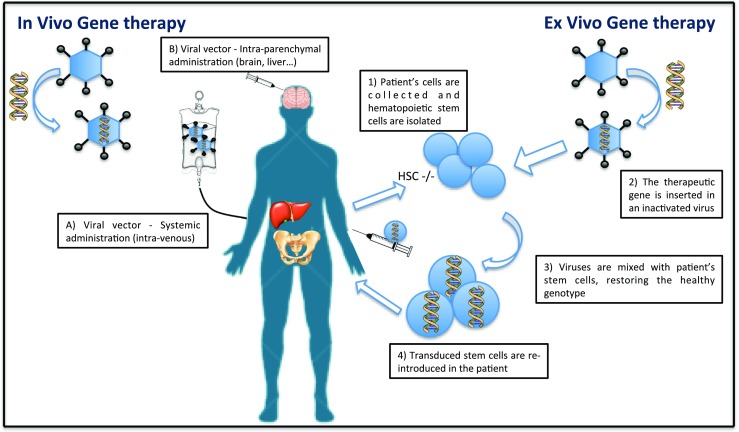
In vivo and ex vivo gene therapy. In both methods, the therapeutic gene is inserted into viral vectors. With in vivo GT, represented on the left of the image, the vectors can be administered through intra-parenchymal or systemic routes. In ex vivo GT (on the right), patient’s cells are collected and stem cells are isolated, which are later mixed with the viral vector. The final transduced stem cells are later re-infused in the patient, restoring the healthy phenotype

Gene therapy is the delivery of genetic material into an individual’s cells and tissues to treat inherited or acquired diseases, or for prophylactic purposes (Kay et al 2001). It is a promising molecular approach that directly delivers into the host cell nucleus a gene aimed to repair the genetic defect in order to cure or ameliorate the clinical phenotype (Morgan and Anderson 1993). Monogenic disorders are the ideal targets for GT (Kumar et al 2016), which has been shown to be effective in treating metabolic diseases (Chandler and Venditti 2016, Gessler and Gao 2016), immunodeficiencies (Aiuti et al 2013, Cicalese et al 2016), eye (Samiy 2014) and coagulation disorders (Manno et al 2006). These disorders, spanning from metabolic to immune syndromes, are characterized by the dysfunction of a single specific gene, where the complete absence or the reduction in activity of the gene product causes the pathological phenotype (Dietz 2010). Thus, the introduction of one or more copies of the healthy gene is able to restore the genetic defect. Gene transfer can be achieved using either in vivo or ex vivo procedure. Ex vivo GT implies the isolation of target cells that are genetically modified in vitro (Fig. 2). Cells are harvested either from donors or patients and they are transduced in vitro with a viral vector to express the therapeutic gene at normal or even supra-normal levels. Subsequently, the gene-corrected cells are infused into the patient where they can proliferate and restore the healthy phenotype.

The in vivo method (Fig. 2) delivers genetic material directly to the patient with a systemic or with an intra-parenchymal, in situ administration, to allow specific organ targeting and sufficient protein concentrations in relevant tissue compartments. This procedure however might lead to inadvertent gene transfer into tissues and cell types that are not proper targets and may elicit immune responses towards the transgene and the vector.

Different viral vectors have been exploited as useful vehicles for gene delivery. The basic concept of viral vectors is to harness the innate ability of viruses to deliver genetic material into the infected cell. The principles for the generation of GT viral vectors are the elimination of the viral toxic and infective functions, without altering the capacity to efficiently infect cells and deliver new transgenes (Escors and Breckpot 2010). The most common DNA viral vectors derive from adenoviruses, herpes simplex viruses and adeno-associated virus (AAV). Retrovirus-based vectors (RVs) contain two copies of a single stranded RNA genome, and they are highly relevant for GT, given their ability to efficiently transduce a wide spectrum of target cells, including haematopoietic cells, and to integrate their genome into the host chromatin. Lentiviral vectors (LVs) are RVs that are based on modifications of human immunodeficiency virus (HIV) and have been exploited for GT. These vectors allow an efficient integration into the host genome (Naldini et al 1996) and are able to incorporate themselves with high efficacy into the host DNA, also in cells that are not actively dividing.

## HSC-Gt

Haematopoietic stem cells (HSCs) give rise to all blood cell lineages, including lymphoid (T and B) and myeloid cells. In adults, HSCs mainly reside in the haematopoietic niche of the bone marrow (BM). In the niche, HSCs are closely associated with stromal cells, which support the maintenance of stem cells and haematopoiesis both by secretion of factors and by cell-cell interactions (Tay et al 2016). In order to generate mature blood cells, HSCs differentiate towards multi-lineage progenitors, which undergo expansion and further commitment steps that restrict their developmental potential. This differentiation process ends with the generation of a terminally differentiated progeny.

In HSC-based GT approaches, HSCs culture and manipulation are essential steps to achieve gene transfer. Furthermore, since the target cells are dividing and long-term expression of the corrective gene in their progeny is required, vectors able to integrate into the host genome, such as RVs and LVs, are necessary.

Furthermore, in order to provide the maximal chance for gene corrected cells to expand, conditioning is needed before infusing HSCs into the patients (Bernardo and Aiuti 2016), either with low dose, sufficient in the case of immune deficiencies, or with myeloablative doses, necessary when high levels of engraftment are required, such as in case of lysosomal storage disorders.

Gamma-RVs require cell division for entrance into the target cell nucleus; since HSCs are mostly quiescent cells, stimulation with cytokines that trigger them into the cell cycle is needed to achieve RV transduction. The occurrence of insertional mutagenesis in clinical trials for primary immunodeficiencies (Fischer et al 2015) have hampered the development of HSC-GT, with notable exception of ADA-SCID, which recently was granted marketing authorization in the European Union (Monaco and Faccio 2016).

Data from 18 patients followed for a median of 7 years showed 100% survival, sustained expression of ADA enzyme in the blood, leading to immune reconstitution and reduction in infections, in the absence of leukemic transformation (Cicalese et al 2016).

There is extensive preclinical evidence that self-inactivating LVs carry a lower risk of insertional mutagenesis and no events of insertional oncogenesis have been detected in HSC-GT clinical trials conducted so far. In addition, LVs have the ability to infecting non-dividing cells (Naldini et al 2016) and allow reaching higher levels of gene marking than gamma-RVs with a shorter ex vivo manipulation, with a positive effect on long-term behaviour of transduced HSCs (Capotondo et al 2007). Another important consideration is that LV HSC-GT has the potential to induce enzyme over-expression in HSC progeny, which may translate into greater therapeutic impact than wild-type HSCT. In fact, pre-clinical studies (Biffi et al 2006, Capotondo et al 2007) have shown a key role of enzyme over-expression in haematopoietic cells and microglia, and suggested a requirement for supra-normal levels of enzyme in these cells to achieve significant therapeutic effects on the nervous system.

So far clinical trials using LV transduced HSCs are ongoing in patients with adrenoleukodystrophy (ALD), metachromatic leukodystrophy (MLD), β-thalassemia, Wiskott-Aldrich syndrome (WAS), severe combined immunodeficiency (SCID) caused by adenosine deaminase (ADA) deficiency (ADA-SCID), X-linked SCID (X1-SCID) and chronic granulomatous disease (CGD) (Cicalese and Aiuti 2015, Aubourg 2016).

## Ex vivo GT for LSDs

In the last 15 years, ex vivo GT has been proposed for various LSDs. In 2000, the first clinical trial with retroviral vector gene therapy was approved for Fabry’s disease (FD). The strategy involved retroviral transduction of autologous mesenchymal stem cells from patients with FD with α-galactosidase A cDNA and implantation via an immunoisolation device. Unfortunately, the protocol was later withdrawn (Ruiz de Garibay et al 2013) and no other clinical trials were started.

**Fig. 3:**
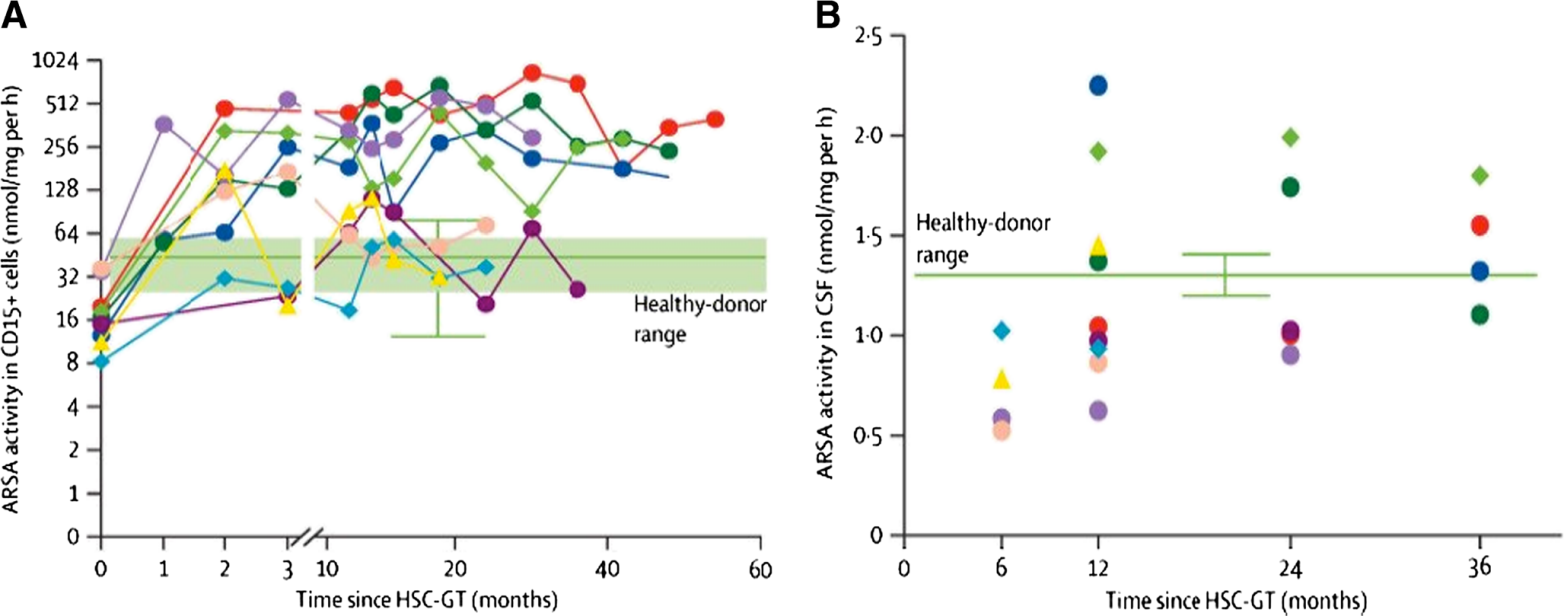
HSC-GT for MLD restores ARSA activity in granulocytes and CSF. (a) ARSA activity measured on peripheral blood CD15^+^ granulocytes. The green box represents the normal activity range collected from six healthy volunteers; (b) ARSA activity measured on CSF of patients at 6, 12, 24 and 36 months follow up. The green horizontal line represents the mean data collected from the activity range measured from three healthy donors (Sessa et al 2016)

In recent years, lentiviral HSC-GT has proven to provide clinical benefit in MLD. After promising results obtained in MLD mouse models (Biffi et al 2004, 2006), a non-randomized, open-label, single-arm phase I/II clinical trial of HSC-GT for the treatment of MLD was started in Milan, Italy, in 2010. The preliminary results on three pre-symptomatic LI children showed stable engraftment of the transduced HSCs in the BM and peripheral blood (Biffi et al 2013), with reconstitution of ARSA activity in all haematopoietic lineages and in the cerebrospinal fluid (CSF). Further results on the first nine treated patients (six LI, two early juvenile and one with an intermediate phenotype) were recently published (Sessa et al 2016), with a median follow up of 36 months (range 18–54 months). These results showed haematopoietic reconstitution in all analysed patients, stable engraftment of gene corrected cells and stable reconstitution of ARSA activity that was also restored in the CSF as early as 6 months after HSC-GT (Fig. 3). The presence of ARSA activity in the CSF represents indirect evidence that HSC-derived cells have migrated to the CNS and produce the enzyme locally. Moreover, this study underlined that the extent of benefit was influenced by the time interval between GT and the expected time of disease onset; in fact, better results were obtained in children treated when pre- or very early-symptomatic, with the maintenance of motor and cognitive functions and the prevention or delay of CNS demyelination.

**Fig. 4:**
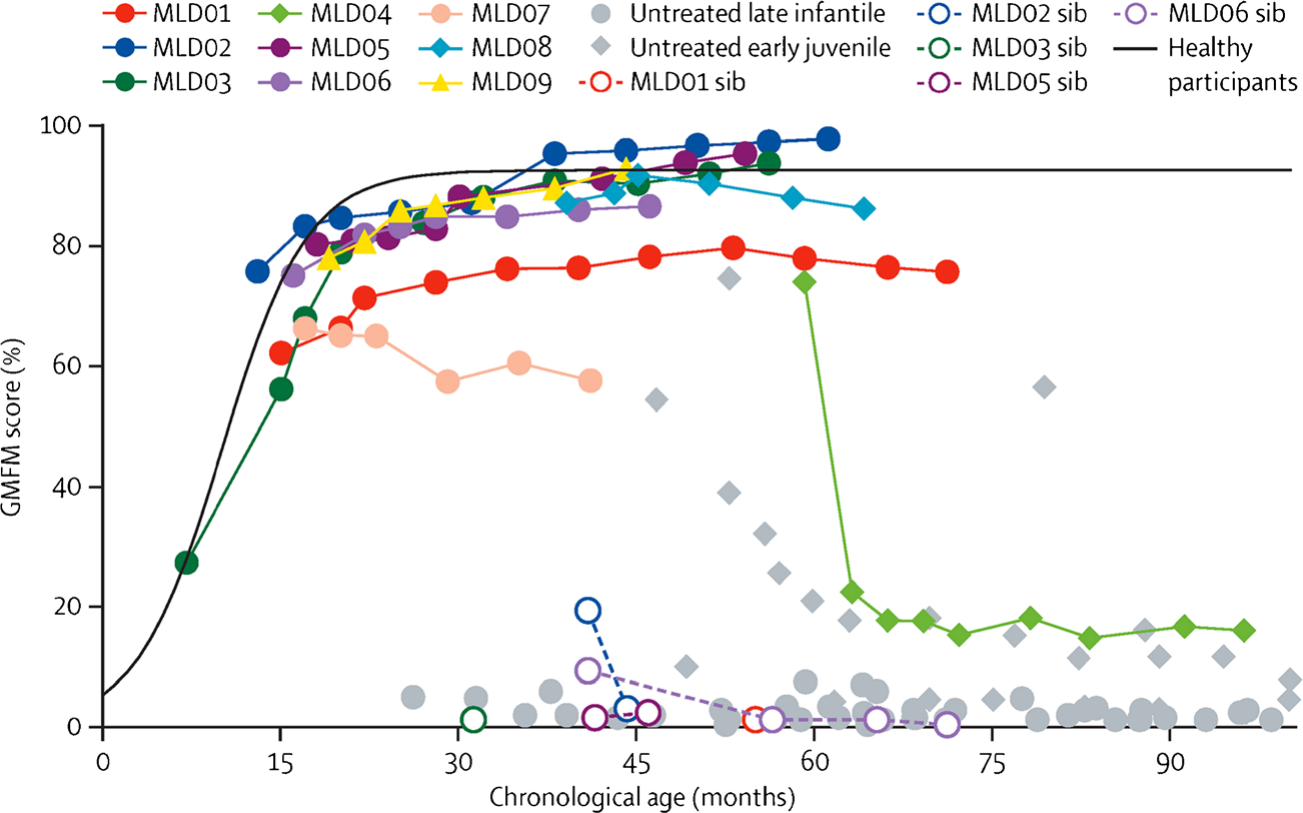
Effect of HSC-GT on motor functions. GMFM scores of patients who underwent HSC-GT (represented with colours) are compared to respective older affected siblings (open circles and dotted lines) and a historical cohort of untreated patients with late-infantile disease (grey circles) and early-juvenile disease (grey diamonds). The black line represents the estimated curve obtained from the scores from 34 healthy participants aged between 0 and 6 years (Sessa et al 2016)

The Gross Motor Function Measurement (GMFM) scores showed that gross motor performance was similar to that of normally developing children for most of the patients who underwent HSC-GT up to the last follow-up (Fig. 4). Apart from one patient, all the other patients who underwent HSC-GT had a progressive increase and/or stabilization in GMFM score during the study, consistent with the acquisition of new motor skills, and/or stability of motor performance compared to the pre-treatment phase. In addition, the same held true for cognitive performances, and all children had a neurocognitive development measured by IQ score within the normal range (except for one patient, who experienced severe disease progression). The patient who showed a rapid motor and cognitive regression was an early juvenile patient who had rapid disease progression after enrolment and was treated when symptomatic.

In regards to CNS involvement, the massive demyelination and severe atrophy observed in untreated patients were not observed in the majority of patients who underwent HSC-GT. The magnetic resonance scores (typically associated with extended white matter involvement, presence and extension of focal and global atrophy and presence of tigroid aspect (Biffi et al 2008)), remained substantially lower than those of the untreated cohort throughout the follow-up. Furthermore, some GT-treated patients displayed a decrease in intensity and extension of white matter signal alteration, suggesting the possibility of CNS remyelination. These studies have shown encouraging outcomes in LI population; a longer follow up will be needed to confirm these data also in a wider EJ population.

Promising results have been obtained in preclinical studies for the treatment of globoid cell leukodystrophy (GLD), which is caused by mutation in the gene encoding galactocerebrosidase (GALC). Gentner et al (Gentner et al 2010) were able to reconstitute enzymatic activity in liver and brains of GLD mice with transplantation of gene-corrected haematopoietic stem and progenitors cells (HSPC), associated to an amelioration of the overall disease phenotype and improvement in survival. Furthermore, they showed how a specific microRNA could down-regulate GALC expression from a genetically modified construct containing the GALC-encoding gene preceded by a microRNA target sequence, protecting HSPCs from transgene toxicity. This allowed a tight regulation of enzyme expression in haematopoietic cells (Visigalli et al 2010) and an important improvement of the disease phenotypes of GCL mice.

Considering the limitations of allogeneic transplantation in MPS-I, HSC-GT has been pursued with the rationale to induce IDUA overexpression in multiple tissues and decrease the residual disease burden. Overexpression of IDUA enzyme in haematopoietic cells and cross-correction of other cell types may be able to correct this defect not only in the CNS but also in the skeleton, which are difficult to target by allogenic HSCT, and able to improve the long-term outcome. In a pre-clinical model of MPS-I, Visigalli et al (Visigalli et al 2010) demonstrated that LV-mediated HSC transfer of IDUA can provide enzyme delivery to all affected tissues, resulting in a complete correction of disease manifestation, including neurologic and skeletal abnormalities. Furthermore, mice who received IDUA-encoding LV (IDUA-LV) transduced cells displayed supranormal enzyme expression both in peripheral blood mononuclear cells (PBMCs), in the serum and in all tested tissues of GT mice. In addition, a recent pre-clinical study (Visigalli et al 2016) analysed toxicology and biodistribution aspects related to IDUA LV-transduced HSPCs in Idua^−/−^ mice: results confirmed absence of toxic or tumorigenic effect of transduced cells, with a sustained engraftment.

Based on these results, there is a strong rationale for implementing GT to correct brain and skeletal defects in patients. A phase I/II clinical trial is under development at SR-TIGET in Milan with the objective to evaluate safety and efficacy of autologous HSC-GT in MPS-I.

## In vivo gene therapy

In vivo GT for LSDs is usually designed to establish a sustained source of therapeutic enzyme within the body for metabolic correction. Intravenous delivery of viral vectors may represent an effective strategy to target gene transfer to tissues, which are readily accessible from the bloodstream and may thus become efficient sources of systemic enzyme distribution. Despite encouraging pre-clinical data on several LSD animal models (Cardone 2007), this approach has revealed important limitations when considering clinical translation: i) the occurrence of immune responses directed towards the vector or the novel therapeutic protein, that can lead to clearance of the transduced cells and/or loss of enzyme activity; ii) poor therapeutic potential in LSDs with CNS involvement, since the secreted enzyme is unable to cross the BBB, thus limiting the benefit to peripheral organs, without correcting nervous system manifestations; iii) the safety and toxicity of parenteral vector administration and the possible risk of inadvertent gene transfer to the gonads and germ-line transmission of the vector.

**Fig. 5:**
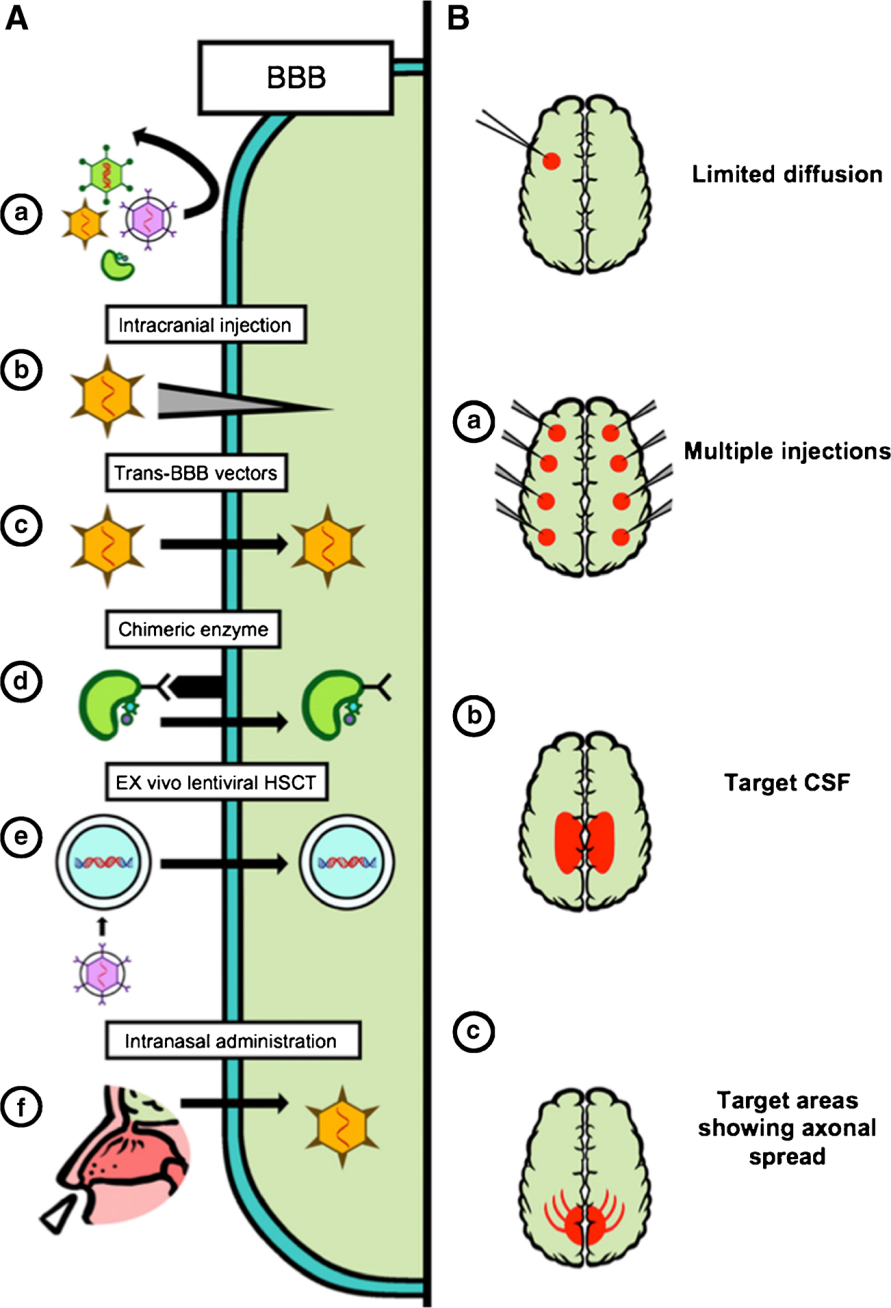
Direct in vivo gene transfer in CNS. A: a) The BBB prevents most viruses and enzymes from entry into the CNS. (b) Intracranial injections (c) Certain AAV can cross the BBB (d) The enzyme can be modified to have affinity for receptors that traffic proteins across the BBB (e) Haematopoietic stem cells can be transfected ex vivo, then reintroduced to the patient. They can cross the BBB, carrying the transfected gene into the CNS. (f) Intranasal virus delivery. B: (a) Multiple injections to target multiple areas (b) CSF target to distribute the virus (c) Target areas of axonal spread (Rastall and Amalfitano 2015)

To overcome the issue of poor BBB permeability for lysosomal enzymes, delivery systems have been developed for direct in vivo gene transfer into the CNS (Rastall and Amalfitano 2015) (Fig. 5). The most deeply investigated strategy consists of the anatomical bypass of the BBB with intracranial injections of the vector. This technique has been exploited in different neuropathic LSDs, such as in infantile neuronal ceroid lipofuscinoses (INCL) a disease caused by mutations in the PPT1 gene that leads to the loss of palmitoyl-protein thioesterase 1 (PPT1). In 2005, the first in vivo GT trial for a LSD was started in New York (Worgall et al 2008), exploiting the injection of serotype 2 adeno-associated virus (AAV2) to transfer normal CLN2 cDNA to children affected by INCL. Ten children with INCL were treated, five of whom were severely affected by the disease and five of whom were moderately affected; each of them received 12 intracranial injections of the AAV2 vector. Treated patients showed at 6 months follow up a slower decline assessed with the modified Hamburg neurological rating scale, but unfortunately the disease progression could not be blocked.

Intra-cerebral delivery of AAV encoding ARSA could be an efficient strategy to rapidly deliver high amounts of ARSA into the brain of symptomatic patients with early onset forms of MLD, even though this approach is not expected to affect PNS disease manifestations. Although brain gene therapy with AAV is expected to target mostly neurons in vivo (Cearley and Wolfe 2006, Sondhi et al 2007), the capacity of lysosomal enzyme to be secreted by transduced neurons and recaptured by other cells opens the possibility to correct oligodendrocytes. The efficacy of this cross-correction likely requires a high number of neurons to be transduced and a high expression of ARSA enzyme (Consiglio et al 2007). Furthermore, AAV vector and ARSA enzyme can be transported along axons, expanding the expression of the therapeutic enzyme within the brain (Luca et al 2005, Chen et al 2006). Proof of concept that intracerebral delivery of AAV5 and lentiviral vectors encoding ARSA is efficient in MLD mice has been achieved (Sevin et al 2007, Lattanzi et al 2010). Efficacy of intra-brain GT using serotype 5 recombinant AAV (AAV5) encoding human *ARSA*, under the control of the PGK promoter, has been demonstrated in ARSA-deficient mice and normal non-human primates (NHPs) (Colle et al 2010).

Different work performed by Sondhi and collegues (Sondhi et al 2005, Sondhi et al 2007, 2012) demonstrated the efficacy of intracerebral administration of AAVrh.10 expressing the lysosomal enzyme tripeptidyl 1-peptiase I (TPP-I) in the brain of NHPs with late infantile neuronal ceroid lipofuscinoses (LINCL). These promising results obtained for LINCL led to different experiments performed with AAV-10 for MLD (Piguet et al 2012). Results showed that AAVrh.10 vector intracerebral injection leads to higher vector genome copies also at a distance from the injection sites, better expression of ARSA enzyme in the brain, detection of ARSA in oligodendrocytes, and biochemical correction of oligodendrocyte-specific sulfatide species. According to these results in NHPs, a phase I/II clinical study to assess safety and efficacy of *ARSA* gene transfer in the brain of children affected with early onset forms of MLD has started in France and is now actively recruiting patients (ClinicalTrials.gov Identifier: NCT01801709) (Aubourg 2016). The study involves the intra-cerebral administration of AAVrh.10cuARSA at 12 sites in the white matter of both brain hemispheres (through six image-guided tracks, with two deposits per track) (Zerah et al 2015). The first two patients received a low dose (1 × 10^12^ gc) while the following three patients received a higher dose of 4 × 10^12^ gc. No results are available yet.

Safety and efficacy of intrathecal delivery of a AAV9 vector expressing IDUA in a feline model of MPS-I have been evaluated in the last years by Wilson’s group (Hinderer et al 2014a, b). Results obtained from these studies showed a rapid elevation in both CSF and serum IDUA activity following vector injection in treated animals. CSF IDUA activity stabilized at approximately normal levels in three cats, showing that intrathecal AAV9 delivery can globally correct MPS-I CNS manifestations. In two animals, after an initial peak post-injection, CSF enzyme levels declined to near baseline in two animals, probably due to the antibodies induction towards IDUA. In the same MPS-I feline model, liver-directed GT (Hinderer et al 2014a, b) was also tested with intravenous injection of an AAV serotype 8 vector expressing feline IDUA from a liver-specific promoter. Three out of four treated animals showed supraphysiologic IDUA expression, GAG clearance from all tissues examined, such as liver, lung, kidney and spleen and reversion of aortic valve lesions. These studies show how this approach may be exploited to also target specific tissues, usually refractory to treatments.

Another promising approach for liver-directed protein replacement therapies is site-specific genome editing, which has been proved to be successful in many pre-clinical models. Sharma et al (Sharma et al 2015) exploited zinc finger nuclease (ZFN) mediated site-specific integration of therapeutic transgenes within the albumin gene by using adeno-associated viral (AAV) vector delivery in vivo. In this experiment, to address LSDs liver damage, wild-type mice were treated with AAV8-ZFN encoding either human α-galactosidase A (Fabry disease), acid β-glucosidase (Gaucher diseases), iduronate-2 sulfatase (Hunter syndrome), or α-l-iduronidase (Hurler’s syndrome). One month after administration, all four lysosomal enzymes were detectable in liver of treated mice, indicating the potential for therapeutic use of genome editing.

Different experiments have also been performed useing LV intracerebral gene delivery. In the last year, the group of Gritti at SR-TIGET studied the use of a single injection of LV.ARSA and LV.GALC in WM of MLD and GLD mice, showing a widespread activity of these enzymes expression in CNS tissue (Lattanzi et al 2010, 2014). Meneghini et al (Meneghini et al 2016) showed how lentiviral vector-mediated intracerebral GT, performed in juvenile non human primates (NHP) affected by MLD and GLD, has a good safety profile, can provide a stable enzyme activity in the whole brain, spinal cord, and cerebro-spinal fluid (CSF) and an efficient gene transfer in neurons, astrocytes and oligodendrocytes close to the injection sites. Furthermore, a robust production and extensive spreading of transgenic enzymes in the whole CNS and in CSF was found, with supra-physiological ARSA activity in normal NHP and close to physiological GALC activity in Krabbe NHP.

In summary, in vivo gene therapy approaches for LSDs have demonstrated satisfactory pre-clinical safety and efficacy to date. The effective correction of disease manifestations throughout large segments of the brain despite the focal vector administration has been shown to be due to the transduction of neurons, which, through their projections, could distribute the enzyme into the CNS (Luca et al 2005, Passini et al 2006). Intra-cerebral delivery of viral vectors encoding *ARSA* has potentially the advantage to allow, at least in the short-term, more rapid and significant expression of ARSA in the brain than HSC-GT with LVs. This aspect could be of great interest, particularly when considering a combined approach of HSC-based GT and in vivo GT, to address concerns related to the therapeutic lag in enzyme deliver to the CNS after HSC-based GT and give the opportunity to restore the functional enzyme as early as possible into the affected brain. Nevertheless, the invasiveness of the gene transfer procedure and the risk of immune reaction against the transgene must not be underestimated for in vivo GT.

## Conclusion

In the last few years, substantial progress has been made in gene therapy for LSDs. Preclinical studies based on ex vivo and in vivo approaches for other diseases have proven to have a significant therapeutic impact on disease outcome. LV-mediated HSC-GT for MLD appears to change the outcome of the disease in LI pre-symptomatic patients. Concerning EJ children, a longer follow up is needed to confirm these data. For this reason, it is of paramount importance to perform an effective therapy before the onset of disease manifestations, which might be enabled through the development of adequate newborn screening assays, such as those based on expanded mass spectrometry (Ombrone et al 2016).

The group of Dr. Gelb (Spacil et al 2016) developed new tandem mass spectrometry methods able to detect sulfatides of MLD patients both in dried blood samples (DBS), with ultra–high-performance liquid chromatography and in urine samples. Both methods revealed important differences between MLD patients and controls, suggesting the feasibility of the mass spectrometry method for implementing newborn screening of MLD. For MPS-I, multiplex-tandem mass spectrometry enzymatic activity assay of IDUA (Elliott et al 2016) has been shown to be an effective method for screening. MPS-I newborn testing has recently been recommended to be included in the Newborn Screening Panel in the US.

Ongoing and future clinical trials will provide essential insights for the treatment and possible cure of LSDs through gene therapy approaches.
